# Structure and reactivity of *Trypanosoma brucei* pteridine reductase: inhibition by the archetypal antifolate methotrexate

**DOI:** 10.1111/j.1365-2958.2006.05332.x

**Published:** 2006-08-10

**Authors:** Alice Dawson, Federica Gibellini, Natasha Sienkiewicz, Lindsay B Tulloch, Paul K Fyfe, Karen McLuskey, Alan H Fairlamb, William N Hunter

**Affiliations:** Division of Biological Chemistry and Molecular Microbiology, School of Life Sciences, University of Dundee Dundee DD1 5EH, UK

## Abstract

The protozoan *Trypanosoma brucei* has a functional pteridine reductase (*Tb*PTR1), an NADPH-dependent short-chain reductase that participates in the salvage of pterins, which are essential for parasite growth. PTR1 displays broad-spectrum activity with pterins and folates, provides a metabolic bypass for inhibition of the trypanosomatid dihydrofolate reductase and therefore compromises the use of antifolates for treatment of trypanosomiasis. Catalytic properties of recombinant *Tb*PTR1 and inhibition by the archetypal antifolate methotrexate have been characterized and the crystal structure of the ternary complex with cofactor NADP^+^ and the inhibitor determined at 2.2 Å resolution. This enzyme shares 50% amino acid sequence identity with *Leishmania major* PTR1 (*Lm*PTR1) and comparisons show that the architecture of the cofactor binding site, and the catalytic centre are highly conserved, as are most interactions with the inhibitor. However, specific amino acid differences, in particular the placement of Trp221 at the side of the active site, and adjustment of the β6-α6 loop and α6 helix at one side of the substrate-binding cleft significantly reduce the size of the substrate binding site of *Tb*PTR1 and alter the chemical properties compared with *Lm*PTR1. A reactive Cys168, within the active site cleft, in conjunction with the C-terminus carboxyl group and His267 of a partner subunit forms a triad similar to the catalytic component of cysteine proteases. *Tb*PTR1 therefore offers novel structural features to exploit in the search for inhibitors of therapeutic value against African trypanosomiasis.

## Introduction

Trypanosomatid protozoans are auxotrophic for folate and other pterins (Kidder and Dutta, 1958; Beck and Ullman, 1990) and have evolved a sophisticated pathway for acquisition and salvage of pteridines from their hosts by relying on a bifunctional dihydrofolate reductase (DHFR; EC 1.5.1.3) – thymidylate synthase (TS; EC 2.1.1.45) together with pteridine reductase (PTR1; EC 1.5.1.33) to carry out reductions of these essential nutrients (Nare *et al*., 1997a). The metabolic pathway that generates reduced folate cofactors is a successful target for the treatment of bacterial infections and some parasitic diseases, notably malaria. This is mainly achieved by inhibition of DHFR (Gilman *et al*., 1990; Blakley, 1995; Kompis *et al*., 2005) and in theory antifolates should provide useful drugs for diseases that result from infection with trypanosomatids. These include diseases such as African sleeping sickness caused by *Trypanosoma brucei gambiense* or *Trypanosoma brucei rhodesiense*. However, the classical inhibitors of folate biosynthesis are ineffective against *Leishmania* and *Trypanosoma*, with resistance mediated through several mechanisms including the amplification of PTR1 (Hardy *et al*., 1997; Nare *et al*., 1997a).

**Fig. 1:**
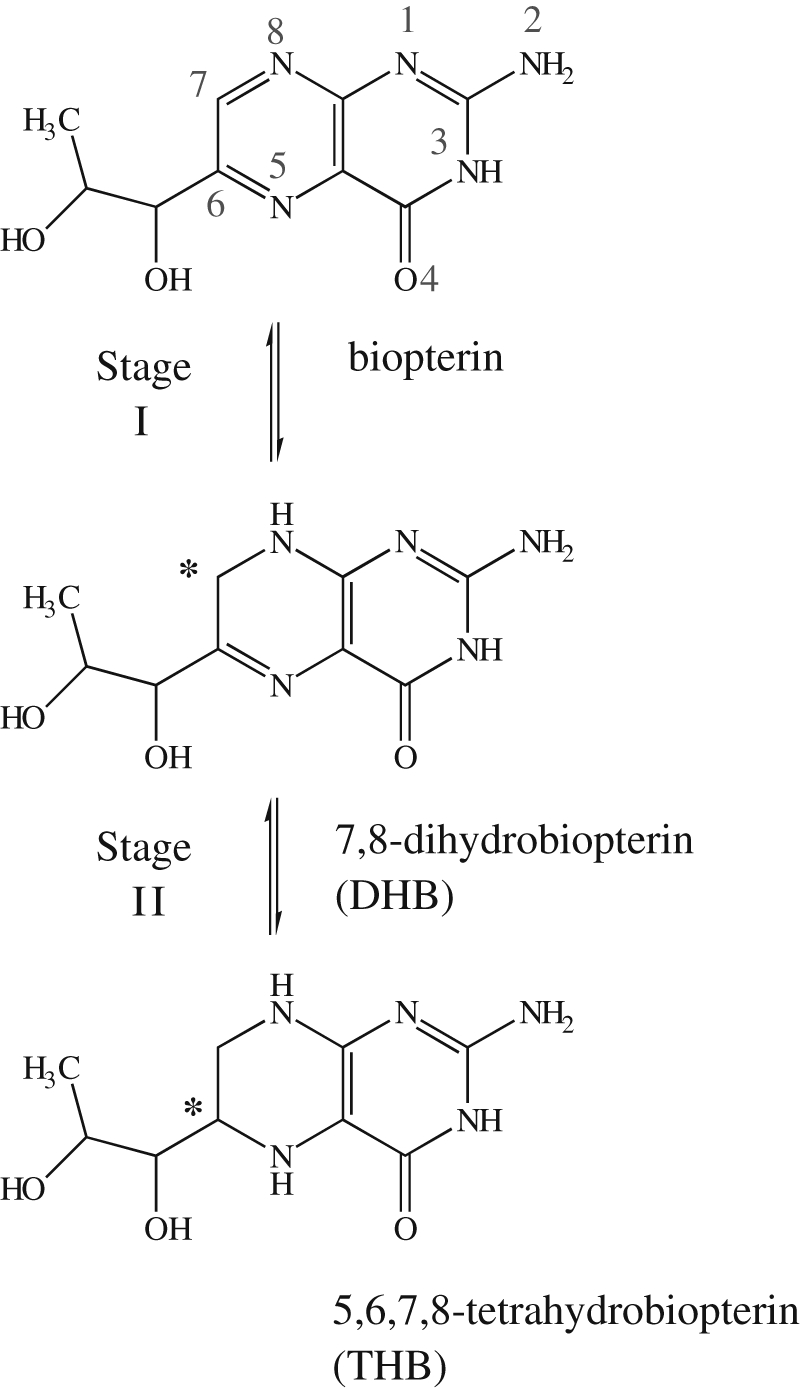
The two-stage reduction of biopterin [2-amino-6-(1,2-dihydroxypropyl) pteridin-4(3*H*)-one] to 7,8-dihydrobiopterin [DHB, 2-amino-7,8-dihydro-6-(1,2-dihydroxypropyl)pteridin-4(3*H*)-one] then to 5,6,7,8-tetrahydrobiopterin [THB, 2-amino-5,6,7,8-tetrahydro-6-(1,2-dihydroxypropyl)pteridin-4(3*H*)-one] catalysed by PTR1. Each stage requires one reducing equivalent provided by the cofactor NADPH and the C atoms (C7 and C6) that accept the hydride are marked with an asterisk (*). This figure and Fig. 2A were prepared using ChemDraw (CambridgeSoft, USA).

PTR1 is a short-chain dehydrogenase/reductase (SDR) able to catalyse the NADPH-dependent two-stage reduction of oxidized pterins to the active tetrahydro-forms (Bello *et al*., 1994; Nare *et al*., 1997b; Luba *et al*., 1998). The enzyme exhibits broad reductase activity, capable of reducing unconjugated (e.g. biopterin) and conjugated (folate) pterins from either the oxidized or dihydro-state (Fig. 1). PTR1 is the only enzyme known to reduce biopterin in *Leishmania* and knockout of the gene indicates that this activity is essential for parasite growth *in vitro* (Bello *et al*., 1994). The biochemical activities of *Leishmania major* PTR1 (*Lm*PTR1) overlap those of DHFR but as PTR1 is less susceptible to inhibition by antifolates it provides a metabolic bypass to alleviate DHFR inhibition (Nare *et al*., 1997a). The *Trypanosoma cruzi* PTR1 homologue (*Tc*PTR1) when overexpressed *in vitro* also leads to antifolate resistance (Robello *et al*., 1997). Gene deletion studies in *T. brucei* have demonstrated that DHFR-TS is essential for growth and null mutants are only able to grow in media supplemented with thymidine. There is also increased resistance to antifolates (N. Sienkiewicz and A.H. Fairlamb, unpubl. data).

**Fig. 2:**
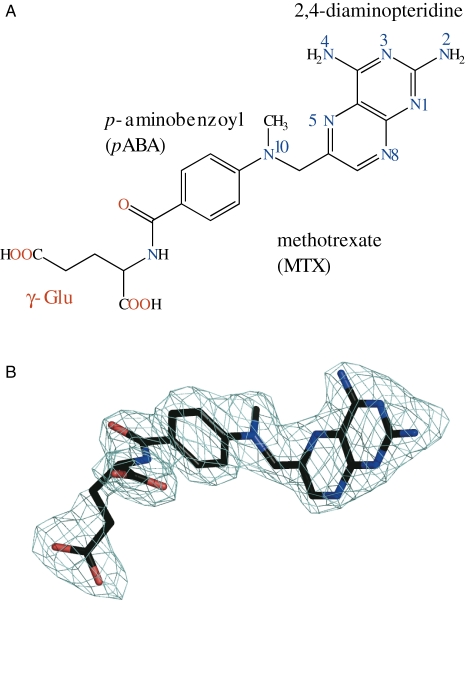
A. The chemical structure of methotrexate (MTX).B. The difference density omit map (chicken wire) for MTX in active site A, calculated with (*F*_*o*_*-F*_*c*_), *α*_*c*_ coefficients and contoured at the 3 σ level. *F*_*o*_ represents the observed structure factors, *F*_*c*_ the calculated structure factors and *α*_*c*_ the calculated phases. The atomic co-ordinates depicted in this figure did not contribute to *F*_*c*_ or *α*_*c*_. MTX in this and subsequent figures is depicted in stick-mode with atomic positions coloured C black, N blue, O red. Figures showing molecular structures were prepared using PyMOL (DeLano, 2002).

The drugs used for treatment of the trypanosomiases are unsatisfactory due to poor efficacy and high toxicity in addition to practical difficulties of administration (Fairlamb, 2003). In principle, the dual inhibition of PTR1 and DHFR activity of trypanosomatids should provide a new therapeutic approach therefore a comprehensive understanding of the structure–activity relationships of the drug targets is required to support the search for such an urgently required therapy. The structure of *L. major* DHFR-TS is known (Knighton *et al*., 1994) and there is an extensive literature on and medical experience on the targeting of DHFR (Gilman *et al*., 1990; Blakley, 1995; Kompis *et al*., 2005).

The kinetics and stereochemical course of the reductions catalysed by *Lm*PTR1 have been studied together with analysis of a library of inhibitors (Hardy *et al*., 1997; Luba *et al*., 1998). We have reported crystal structures of *Lm*PTR1 in ternary complexes with cofactor, substrates and products [biopterin, 2-amino-7,8-dihydro-6-(1,2-dihydroxypropyl)pteridin-4(3*H*)-one (DHB), 2-amino-5,6,7,8-tetrahydro-6-(1,2-dihydroxypropyl)pteridin-4(3*H*)-one (THB)], and with inhibitors including methotrexate (MTX, 4-amino-*N*10-methyl-pteroylglutamic acid; Fig. 2A; Gourley *et al*., 2001; McLuskey *et al*., 2004; Schüttelkopf *et al*., 2005). Other laboratories have reported structures of PTR1 from *L. tarentolae* (Zhao *et al*., 2003) and PTR2, an isoform from *T. cruzi* (Schormann *et al*., 2005).

We set out to confirm the assignment of a functional PTR1 in *T. brucei* (*Tb*PTR1) and now report on the reactivity and inhibition of this enzyme compared with that derived from other trypanosomatid parasites. Accurate molecular details are critical to support a structure-based approach to inhibitor design and we have determined the crystal structure of the ternary complex with NADP^+^ and MTX. Comparisons of PTR1 from different species allow us to investigate the feasibility of developing a broad-spectrum PTR1 inhibitor or to see whether the enzyme from a specific trypanosomatid species might provide distinct and new opportunities for inhibitor design.

## Results and discussion

### Enzyme activity: *T. brucei* possesses a functional PTR1

A candidate gene for *Tb*PTR1 was cloned, the recombinant protein produced in *Escherichia coli* purified in high yield, of greater than 30 mg of enzyme from each litre of culture, and proven to be enzymatically active. The optimum pH for reduction of biopterin and DHB by *Tb*PTR1 is 3.7 with specific activities of 2.0 and 2.3 μmol min^−1^ mg^−1^ protein respectively. *Tb*PTR1 displays a uniformly low level of activity with respect to reduction of folate and dihydrofolate (DHF), in each case with maximum specific activity about 0.1 μmol min^−1^ mg^−1^.

For reasons that are not understood, PTR1 from different species display different activities. *Tb*PTR1 is equally active with biopterin and DHB as substrates but relatively insensitive to folate and DHF. *Tc*PTR1 is more active with biopterin and folate than with DHB or DHF (Robello *et al*., 1997) while the isoform *Tc*PTR2 is only active against reduced pterins, DHB and DHF (Schormann *et al*., 2005).

*Tb*PTR1 is more active than *Lm*PTR1 with respect to reduction of pterins and the enzymes have different pH optima. For *Lm*PTR1 the pH optimum is at 4.7 compared with 3.7 for *Tb*PTR1. *Tb*PTR1 displays substrate inhibition with DHB as variable substrate as reported for *Lm*PTR1 (Nare *et al*., 1997b). The apparent kinetic constants for DHB are similar: *K*_m_ = 10.9 (±2.4) versus 7.6 μM and 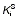 = 3.8 (±0.7) versus 14.5 μM for *Tb*PTR1 and *Lm*PTR1 respectively. However, *V*_max_ is approximately 10-fold higher for the *T. brucei* enzyme (9.1 ± 1.2 U mg^−1^) compared with *Lm*PTR1 (0.87 U mg^−1^) yielding *k*_cat_ = 4.7 s^−1^ and *k*_cat_/*K*_m_ = 4.3 × 10^5^ M^−1^ s^−1^ for *Tb*PTR1 and *k*_cat_ = 0.44 s^−1^ and *k*_cat_/*K*_m_ = 5.8 × 10^4^ M^−1^ s^−1^ for *Lm*PTR1. In contrast to *Lm*PTR1, *Tb*PTR1 appears to be at least fourfold less efficient in reducing folate and DHF over the pH range 4–8. MTX, a molecule of similar shape and mass to folate (Fig. 2), inhibits *Tb*PTR1 less well than it does to *Lm*PTR1. The *K*_i_ for MTX inhibition of *Lm*PTR1 is reported as 58 (±15) nM (Nare *et al*., 1997b) and in our hands 39 (±19) nM, which is a good agreement. The *K*_i_ is 152 (±16) nM with respect to *Tb*PTR1. The slower catalysis of folates and weaker inhibition displayed by MTX against *Tb*PTR1 may be due to the presence of a less flexible and restricted binding pocket in that enzyme compared with *Lm*PTR1 (see active site description below).

### Comments on the crystallographic model

The structure of *Tb*PTR1 has been determined to 2.2 Å resolution and statistics are presented in Table 1. The stereochemistry of the model is acceptable as judged by standard criteria and the fit of the model to the electron density is good. As an example, Fig. 2B shows the omit difference density map associated with a MTX molecule. The asymmetric unit consists of a homotetramer, which represents the functional unit, and overlays of the 251 residues common to each of the four subunits show a root mean square deviation (r.m.s.d.) range in Cα positions of 0.19–0.31 Å (mean 0.25 Å). Visual inspection confirms this high degree of structural conservation. This consistency, without the use of non-crystallographic symmetry (NCS) restraints, indicates that it is only necessary to detail one subunit and one enzyme active site, arbitrarily chosen as subunit A.

**Table 1:** Data collection, refinement and model geometry statistics.

Resolution range	20–2.2 Å
No. of measurements/unique reflections	139 124/50 048
Redundancy/completeness (%)	2.8/99.2 (93.1)^a^
<*I/*σ(*I*)>	12.4 (3.5)^a^
*R*_merge_ (%)^b^	4.6 (15.0)^a^
Wilson *B* (Å^2^)	24.2
Protein residues (total)	1024
In subunits A–D	253, 254, 260, 257
Additional groups	
Solvent/NADP^+^/MTX/Ni^2+^/acetate/cacodylate	801/4/4/2/2/8
*R*_work_ (%)^c^/No. of reflections	15.35/47 369
*R*_free_ (%)^d^/No. of reflections	22.3/2534
Average isotropic thermal parameters (Å^2^)	
Subunits A–D	23.1, 22.6, 23.9, 24.8
NADP^+^	18.7, 17.8, 18.0, 18.9
MTX pteridine	19.8, 16.0, 17.0, 16.8
MTX *p*ABA	23.6, 21.6, 23.8, 26.1
MTX γ-Glu	33.0, 31.5, 38.6, 38.7
Solvents/dimethyl arsinoyl moietyr.m.s.d. bond lengths (Å)/angles (°)	33.20.013/1.402
DPI^e^	0.208
Ramachandran analysis (%)	
Favoured and allowed regions	99.9
Generously allowed regions	0.1 (Arg14 in each subunit)

a. Values in parentheses refer to the highest resolution bin approximately 2.3–2.2 Å.

b. R_merge_ = Σ_h_Σ_i_|I(h,i) – <I(h)>|/Σ_h_Σ_i_ I(h,i), where I(h,i) is the intensity of the ith measurement of reflection h and <I(h)> is the mean value of I(h,i) for all i measurements.

c. R_work_ = Σ_hkl_||F_o_| − |F_c_||/Σ|F_o_|, where F_o_ is the observed structure-factor amplitude and F_c_ the structure-factor amplitude calculated from the model.

d. R_free_ is the same as R_work_ except only calculated using a subset, 5%, of the data that are not included in any least squares refinement calculations.

e. DPI, diffraction-component precision index (Cruickshank, 1999).

### Overall structure

**Fig. 3:**
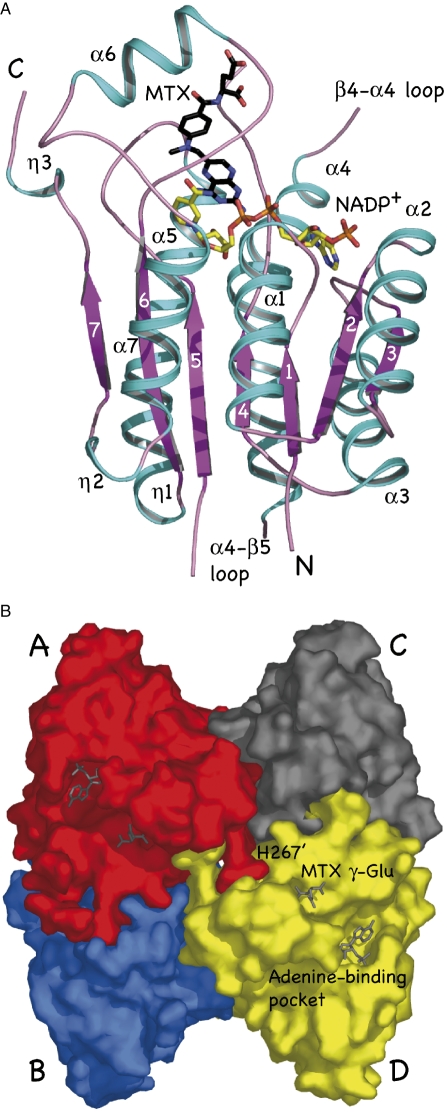
A. Ribbon diagram of the *Tb*PTR1 subunit showing the fold and position of MTX and cofactor. Helices are coloured cyan and labelled α or η (3_10_), β-strands are purple and numbered. Cofactor bonds are drawn as sticks coloured according to atom type; C yellow, N blue, O red, P orange. B. Surface representation of the functional tetramer with individual subunits coloured red, blue, grey and yellow. The cofactor adenine and MTX γ-Glu moieties associated with subunits A and D are depicted as grey sticks. The view is parallel to a twofold axis of NCS. His267′ is labelled to identify the incursion of the C-terminus of one subunit into the active site cleft of an adjacent subunit.

The *Tb*PTR1 subunit forms a single α/β-domain constructed around a seven-stranded parallel β-sheet sandwiched between two sets of α-helices (Fig. 3A). This structure is typical of the SDR superfamily (Duax *et al*., 2003; Oppermann *et al*., 2003). The functional tetramer displays point group 222 (Fig. 3B), with two active sites, separated by approximately 25 Å, on each side of the assembly. Subunit A participates in extensive interactions with subunits B and C. The interface formed with subunit D covers a much smaller area and involves the C-terminus of each subunit placed between the β5-α5 loop and C-terminus of the partner subunit. This interface places a basic residue, Arg287 or His267 in *Lm*PTR1 and *Tb*PTR1, respectively, of one subunit near to the catalytic centre of the partner subunit. This will be discussed later.

**Fig. 4:**
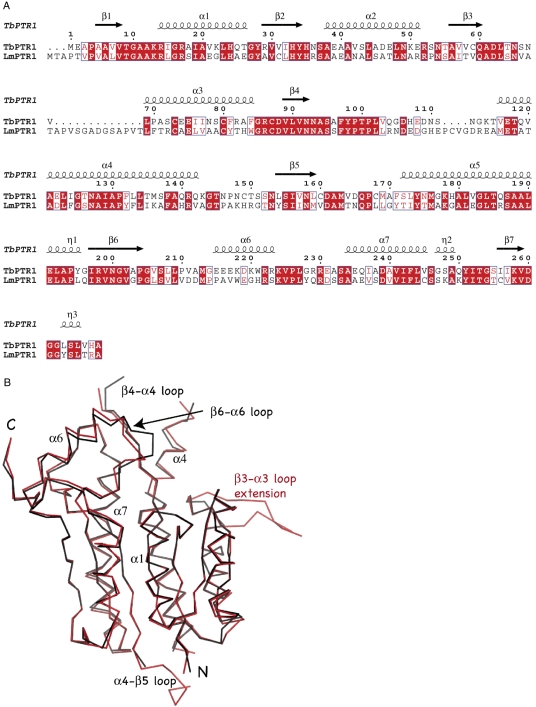
A. The primary and secondary structure for *Tb*PTR1 together with the aligned sequence of *Lm*PTR1. Residues shown in white on a red background are strictly conserved; conservative substitutions are shown in red on a white background. This panel was prepared using ESPript (Gouet *et al*., 1999). B. A Cα trace showing the overlap of a subunit of *Tb*PTR1 (black) and *Lm*PTR1 (red). The view is similar to that used in Fig. 3A.

A structure-based sequence alignment of *Tb*PTR1 and *Lm*PTR1, the sequences share 51% identity, is shown in Fig. 4A. The *Tb*PTR1 topology is closely related to *Lm*PTR1; an overlay of one monomer of *Tb*PTR1 onto one subunit of *Lm*PTR1 matches 244 residues with an r.m.s.d. of 0.71 Å (Fig. 4B). The *Tb*PTR1 sequence is shorter than *Lm*PTR1 due to two deletions and a truncation at the N-terminus. In *Tb*PTR1 a short β3-α3 loop is well ordered whereas in *Lm*PTR1, the loop is extended by 13 residues and generally disordered (Schüttelkopf *et al*., 2005). A second, smaller deletion of four residues occurs at the C-terminal segment of the loop linking β4 and α4 in *Tb*PTR1. This loop is also surface-exposed and disordered in both *Tb*PTR1 and *Lm*PTR1.

The sequence and structure alignments indicate a strong conservation of sequence in elements of secondary structure, in sections of the protein involved in cofactor binding and the catalytic centre. A notable exception concerns the β6-α6 loop and α6 itself, which are placed adjacent to the catalytic centre, and will be discussed below.

### The cofactor binding site and catalytic centre

**Fig. 5:**
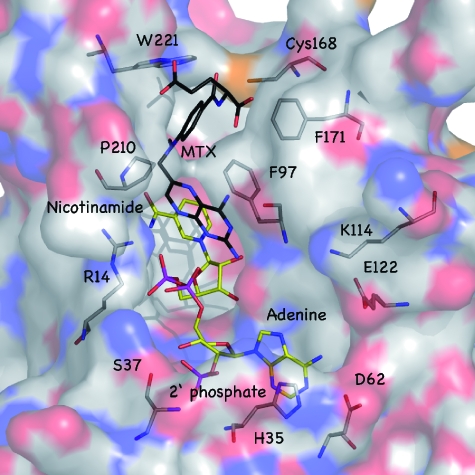
The active site of *Tb*PTR1. The enzyme surface is shown as a transparent van der Waals surface coloured C grey, N blue, O red, S, orange. The cofactor, MTX and selected amino acids are depicted in stick mode.

**Fig. 6:**
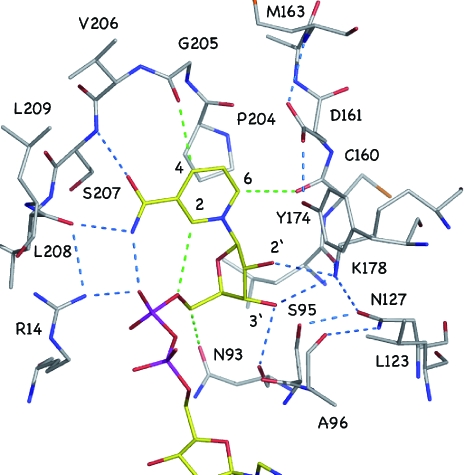
The nicotinamide binding site and selected hydrogen bonds. Conventional hydrogen bonds are shown as blue dashed lines, C–HO interactions by green dashed lines. 2′ and 3′ identify the hydroxyl groups of the nicotinamide ribose and 2, 4, 6 the C atoms of the pyridine moiety. Leu159 is placed below the cofactor ribose and for reasons of clarity is not labelled.

The PTR1 active site is an L-shaped depression nearly 30 Å in length, 22 Å wide and 15 Å deep, formed by the C-terminal ends of the β-sheet (Fig. 3A), where the cofactor binds in an extended conformation. The catalytic centre is created by residues from the C-terminal section of β4 and α5, the two loops between β5-α5 and β6-α6 together with the nicotinamide. Figure 5 shows the cofactor-binding cleft, with selected residues and details of nicotinamide binding depicted in Fig. 6. The pattern of hydrogen bonds formed by SDR family members and cofactors is, in general, well conserved (Duax *et al*., 2003) and such interactions are listed in Table 2.

**Table 2:** Potential hydrogen-bonding contacts (≤3.5 Å) formed by the cofactor (NADP^+^) and inhibitor MTX in TbPTR1 active site A.

Cofactor atom	Partner	MTX atom	Partner
Adenine N6	OD1 Asp62	Pteridine N1	α-Phosphate O2P
Adenine N6	Water	Pteridine N2	OG Ser95
Adenine N7	Water	Pteridine N2	O Ser95
Adenine N1	N Leu63	Pteridine N3	Nicotinamide O2'
Adenine O2′	Water	Pteridine N4	OH Tyr174
Adenine O1P	N Ser37	Pteridine N4	Water
Adenine O1P	N His35	Pteridine N5	Water
Adenine O1P	Water	Pteridine N8	Water
Adenine O2P	OG Ser37	*p*ABA O	Water
Adenine O2P	Water	*p*ABA N	Water
Adenine O2P^a^	Adenine O3'	γ-Glu O1	Water
Adenine O3P	N Asn36	γ-Glu O2	Water
Adenine O3P	Two waters	γ-Glu N	Water
Adenine ribose O3′^a^	Adenine O2P	γ-Glu OE1	Water
Adenine ribose O3′	Two waters	γ-Glu OE2	Water
α-Phosphate O1P	Water		
α-Phosphate O2P	N1 MTX pteridine		
a-Phosphate O2P	Two waters		
β-Phosphate O1P	N Ile15		
β-Phosphate O1P	Water		
β-Phosphate O2P	NH1 Arg14		
β-Phosphate O2P^a^	Nicotinamide N7		
β-Phosphate O5^a^^b^	Nicotinamide C2		
Nicotinamide ribose C5^b^	OD1 Asn93		
Nicotinamide ribose O3′	NZ Lys178		
Nicotinamide ribose O3′	O Asn93		
Nicotinamide ribose O3′	Water		
Nicotinamide ribose O2′	NZ Lys178		
Nicotinamide ribose O2′	N3 MTX pteridine		
Nicotinamide C2^a^^b^	β-Phosphate O5		
Nicotinamide C4^b^	O Gly205		
Nicotinamide C5^b^	Water		
Nicotinamide C6^b^	O Cys160		
Nicotinamide O7	N Ser207		
Nicotinamide N7	O Leu208		
Nicotinamide N7^a^	β-Phosphate O2P		

a. Intramolecular interaction.

b. C-H···O interactions.

The adenine moiety is placed in a well-defined cleft formed by contributions from residues in β1, β2, β3, β4 and α4, sandwiched between a hydrophobic bed created by three side-chains, Leu63, Ala94 and Thr126 (not shown), and the aromatic side-chain of His35. Four hydrogen bond associations involve the adenine 2′ phosphate and likely contribute significantly to cofactor binding. In general, two basic residues, an arginine and lysine pair, bind this phosphate and are recognized as a principal factor in discrimination for NADPH utilization over NADH in the SDR family (Duax *et al*., 2003). This is not the case in *Tb*PTR1, with specificity provided by a phosphate-binding pocket formed mainly by the turn between β2 and α2, the use of main-chain amide groups, contributed from His35, Asn36, Ser37 and the side-chain hydroxyl of Ser37. The corresponding residues in *Lm*PTR1 are His38, Arg39 and Ser40. Hydrogen-bonding interactions with the adenine component of the cofactor are highly conserved between *Tb*PTR1 and *Lm*PTR1 with one exception. In *Lm*PTR1, the adenine N6 amino group donates a hydrogen bond to the side-chain carboxylate group of Asp142. This residue type is conserved in *Tb*PTR1, Glu122, but the side-chain adopts a different orientation to interact with the nearby Lys114 thereby removing a hydrogen bond with the adenine N6 (Fig. 5).

Residues contributed from β1, β4, β5 and the β6-α6 loop create the nicotinamide binding site with the pyridine nucleotide placed over Ile15 (not shown), Pro204 and Ser207, and stacked under the MTX pteridine. The pyridine adopts a *syn* conformation with respect to the ribose and an intramolecular hydrogen bond is formed between N7 and the β-phosphate (Fig. 6).

Kinetic studies suggest an ordered ternary complex mechanism for PTR1, with NADPH binding first and NADP^+^ dissociating after the reduced pteridine product vacates the active site (Luba *et al*., 1998). Crystallographic analyses of ternary complexes of *Lm*PTR1 with the substrates and products, biopterin, DHB and THB, show the pterin ligands in a single orientation participating in virtually identical interactions with the enzyme and cofactor (Schüttelkopf *et al*., 2005). These structures define a sequential two-step reduction mechanism (Fig. 1) and the roles played by three residues, Asp181, Tyr194 and Lys198 in *Lm*PTR1, which in the first catalytic step resembles that proposed for other SDR family members. These residues are conserved in *Tb*PTR1 (Asp161, Tyr174 and Lys178; Fig. 6) and serve to position the nicotinamide of the cofactor for hydride transfer (Lys178), acquire a proton from solvent (Asp161) and pass this proton on to the substrate (Tyr174). The second reduction step, which occurs on the opposite side of the pterin, is similar to that postulated for DHFR. Nicotinamide again provides a hydride and activated water supplies the proton (Gourley *et al*., 2001).

**Fig. 7:**
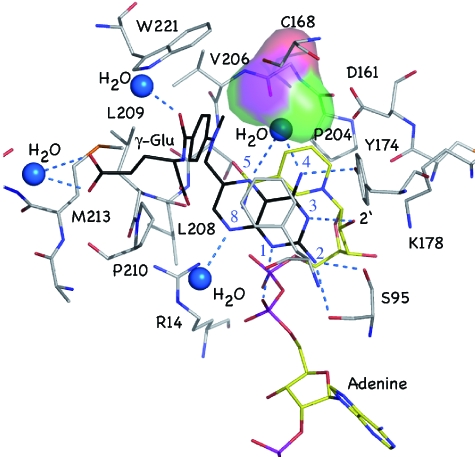
Hydrogen bond interactions formed by MTX with *Tb*PTR1 and cofactor. The van der Waals surface of cacodylate modified Cys168 is shown and coloured purple for As, green for C and red for O. Blue numbers mark the N atoms of the MTX pteridine group. Phe97 is not labelled.

Hydrogen-bonding interactions involving the Asp–Lys–Tyr triad and neighbouring residues (Fig. 6) position the nicotinamide, create the PTR1 catalytic centre and directly influence the enzyme's reactivity. Lys178 participates in four hydrogen bonds with Asn127, Leu159 and both hydroxyl groups of the nicotinamide ribose. The side-chain of Asp161 forms hydrogen-bonding interactions with Tyr174 on one side and Met163 amide on the other. Asp161, a key catalytic residue, by association with Tyr174 facilitates protonation of substrate in the first stage of the enzyme mechanism. The decrease in enzyme activity as pH is raised likely corresponds to deprotonation of Asp161. The cofactor forms two hydrogen bonds with MTX via the α-phosphate and nicotinamide O2′ (Fig. 7). There are five putative C-H**···**O hydrogen bonds, four shown in Fig. 6. These involve the nicotinamide interacting with a β-phosphate oxygen, the carbonyl groups of Cys160 and Gly205, and side-chain of Asn93. The remaining interaction, not shown, involves C5 and a water molecule. These C-H**···**O interactions are weak (Leonard *et al*., 1995) but contribute to the association of protein with cofactor, help to align the nicotinamide to facilitate hydride transfer from C4 and may even contribute to the formation of the transition state during catalysis.

The cofactor binding motif of most SDR family members, GlyX_3_GlyXGly, where X is any amino acid type (Duax *et al*., 2003), is replaced in PTR1 by GlyX_3_ArgXGly, with the arginine side-chain (Arg14 in *Tb*PTR1, Arg17 in *Lm*PTR1) interacting directly with the NADP^+^ pyrophosphate. This interaction, together with two hydrogen bonds donated to the carbonyl groups of Leu208 and Leu209 (only the former is depicted in Fig. 6) forces the main-chain to adopt an unfavourable conformation; Arg14 is the only residue that displays a generously allowed φ/ψ combination in the Ramachandran plot.

There is a significant difference between *Tb*PTR1 and *Lm*PTR1 with respect to nicotinamide binding by the β6-α6 loop, which is also involved in MTX binding. In *Tb*PTR1, the nicotinamide N7 and O7 groups interact with the main-chain carbonyl group of residue Leu208 and the main-chain amide of Ser207 (Fig. 6). In *Lm*PTR1, the polypeptide chain at the C-terminal segment of β6 adopts a different conformation allowing both N7 and O7 to interact with the main-chain atoms of the conserved serine (Ser227; not shown).

### MTX binding

The electron density for MTX in complex with *Tb*PTR1 is well defined over the entire molecule (Fig. 2B) in contrast to the structures of *Lm*PTR1 and *Tc*PTR2, where the γ-Glu tail is poorly defined (Gourley *et al*., 2001; Schormann *et al*., 2005) and *Lt*PTR1 where the inhibitor was not located despite it being included in the crystallization solution (Zhao *et al*., 2003).

The association between *Tb*PTR1/NADP^+^ and MTX is primarily through interactions with the pteridine moiety, which is sandwiched between the nicotinamide and Phe97. This phenylalanine forms a hydrophobic region at the edge of the catalytic centre by association with Phe171 (Fig. 5) and Tyr177 (not shown). All functional groups of MTX participate in hydrogen-bonding interactions with the enzyme and the cofactor, either directly or via solvent-mediated hydrogen-bonding networks (Fig. 7, Table 2). Four of the six N atoms of the pteridine moiety make direct interactions: N1 with the α-phosphate group of the cofactor and is likely protonated, N2 donates hydrogen bonds to the side-chain hydroxyl and main-chain carbonyl groups of Ser95, N3 accepts a hydrogen bond from the 2′ hydroxyl group of the nicotinamide ribose. The amino N4 donates two hydrogen bonds to Tyr174 and a water molecule. The remaining two N atoms, N5 and N8, interact with water molecules. The former accepts a hydrogen bond from the N4-bound water, which also associates with the modified Cys168 and another water that links with the side-chain of Asp161 (not shown). N8 interacts with a water molecule that in turn associates with the cofactor α-phosphate and other waters. A noteworthy similarity between PTR1 and DHFR is that the pteridine moiety of MTX binds in a different orientation to that adopted by substrates (Charlton *et al*., 1985; Matthews *et al*., 1985; Gourley *et al*., 2001) with the pteridines rotated about the N2–N5 axis by 180° relative to each other. This difference results from each ligand adopting a specific orientation to satisfy and maximize hydrogen-bonding capacity.

The *para*-aminobenzoic acid (*p*ABA) and γ-Glu components of MTX are directed out of the binding site with the γ-Glu tail of MTX directed towards the N-terminus of the β6-α6 loop. Hydrogen-bonding interactions are formed only with water molecules, two of which also interact with functional groups on the enzyme. A well-defined water links the *p*ABA carbonyl with NE1 of Trp221 and another links γ-Glu OE1 and OE2 to the amide of Gly214 (not shown). The *p*ABA moiety is positioned with Phe97 and Phe171 on one side, Met213 and Trp221 on the other. The methyl substituent at N10 participates in van der Waals interactions with the side-chain of Val206 and with a dimethylarsinoyl-modified Cys168. Cacodylate was used as the buffer in the crystallization mixture and this will be discussed below.

**Fig. 8:**
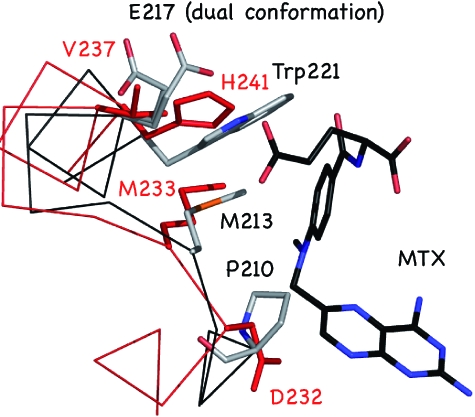
Overlay Cα trace of the β6-α6 loops and part of the helices of *Tb*PTR1 (black) and *Lm*PTR1 (red). Side-chains for *Lm*ptr1 residues are shown as red sticks. Both conformers of *Tb*PTR1 Glu217 are shown.

The β6-α6 loop is well defined in *Tb*PTR1 and adopts a similar conformation in all subunits. In contrast, in *Lm*PTR1, this loop is flexible and adopts different conformations. In this section of PTR1 the sequence homology between the *L. major* and *T. brucei* enzymes is poor (Fig. 4A) and differences are observed (Fig. 8). For example, in *Lm*PTR1 Asp232 interacts with Arg17, which is involved in cofactor binding. The arginine is conserved in both structures (Arg14 in *Tb*PTR1) but this interaction is removed as the residue that corresponds to *Lm*PTR1 Asp232 is *Tb*PTR1 Pro210 (Fig. 8). The position of α6 observed in *Tb*PTR1 places Met213 and Trp221 closer to the *p*ABA group compared with the corresponding residues, Met233 and His241, of *Lm*PTR1 (Fig. 8). The presence of the larger tryptophan side-chain at this position in *Tb*PTR1 compared with a histidine in *Lm*PTR1, or tyrosine in both *Tc*PTR2 and *Lt*PTR1 in conjunction with the α6 adjustment reduces the size of the *p*ABA binding region in the enzyme derived from the African trypanosome and introduces a significant chemical change in this area of the active site.

The quaternary structure places the C-terminal section of one subunit close to the active site of a partner subunit. In *Lm*PTR1, a basic residue, Arg287 is directed towards and linked through solvent to the catalytic centre (Gourley *et al*., 2001). In *Tb*PTR1 the C-terminal basic residue is His267 but the presence of Trp221 in conjunction with a chemically modified Cys168 (the corresponding residue is Leu184 in *LmPTR1*) occludes the presence of a solvent network in this part of the active site. The modification of Cys168 by reaction with cacodylate buffer to form dimethylarsinoyl cysteine is intriguing but not unusual in protein chemistry and it is uncertain whether such reactivity has a biological function in *T. brucei*. The arrangement of the C-terminus carboxylate, the penultimate residue His267, and Cys168 of the partner subunit is similar to that observed for the catalytic triad of cysteine proteases (Tyndall *et al*., 2005). Such an arrangement may enhance the reactivity of the cysteine. One other solvent-exposed residue, Cys59, is modified to dimethylarsinoyl cysteine. Nearby, < 5 Å distance, is His33, which may also help to activate that thiol group. Examples of omit difference density maps for the modification of Cys59 and Cys168 of subunit A are shown in Fig. S1 in *Supplementary material*.

The presence of dimethylarsinoyl in the active site, a consequence of using cacodylate buffer, could compromise kinetic characterization therefore assays were carried out in citrate, phosphate and acetate buffers. Mass spectrometry characterization of freshly purified sample was consistent with an unmodified protein (data not shown) and recently, isomorphous crystals of *Tb*PTR1 were obtained in 10 mM sodium citrate buffer and the high-resolution structure indicates that both Cys59 and Cys168 are not modified (unpublished data).

### Concluding remarks: the stage is set for structure-based inhibitor design

Combinations of drugs, each displaying independent modes of action, can improve efficacy in antimicrobial treatments without increasing toxicity and with the added benefit of providing some protection against the development of drug resistance. Examples of therapeutically useful combinations include dapsone with chloroproguanil or pyrimethamine to combat malaria (Olliaro and Taylor, 2003). The principle is well established and compatible with a strategy that involves targeting two enzyme activities, those of DHFR and PTR1, towards the goal of developing new treatments for trypanosomatid infections. Either a single molecule that is a potent inhibitor of both enzymes, or two compounds specific for each are required. A number of compounds active against both enzymes have been characterized but in these cases either PTR1 is less susceptible to inhibition than DHFR or the level of inhibition is poor (Hardy *et al*., 1997; Schüttelkopf *et al*., 2005) hence such molecules lack efficacy against trypanosomatids. Pyrimethamine is a highly potent inhibitor of *T. brucei* DHFR and would constitute a suitable drug partner to be combined with a specific novel inhibitor of PTR1 (Jaffe *et al*., 1969; Sirawaraporn *et al*., 1988).

Despite being able to catalyse the same reaction, DHFR presents distinct structural features compared with PTR1 allowing it to bind cofactor and substrate or inhibitors in any order, although with a kinetic preference. DHFR also undergoes extensive conformational changes upon ternary complex formation (Sawaya and Kraut, 1997; Schnell *et al*., 2004), whereas PTR1 appears more rigid (Schüttelkopf *et al*., 2005). DHFR exhibits much stronger interactions, electrostatic and hydrophobic, with the *p*ABA-γ-Glu group of various ligands than PTR1 while the latter enzyme presents a sterically restricted catalytic centre, in particular with the presence of a phenylalanine (Phe97 in *Tb*PTR1) that associates intimately with the pteridine. These differences render it extremely difficult to envisage a single compound with the necessary inhibitory properties for use against both DHFR and PTR1. There are already potent DHFR inhibitors with well-characterized pharmacokinetics (Kompis *et al*., 2005) and we conclude that the priority must be development of PTR1 inhibitors to complement existing drugs.

The structural similarity of PTR1/PTR2 from different species suggests that inhibitors are likely to have broad-spectrum activity. However, the active site of *Tb*PTR1 offers distinct features of interest. First, the presence of Trp221 near the substrate binding site provides a valuable feature to factor into inhibitor design with excellent potential to exploit the side-chain and enhance hydrophobic interactions with inhibitors. While this opportunity could greatly assist the development of a tight binding PTR1 inhibitor such a molecule may only be sufficiently potent against *Tb*PTR1. Given that the most serious need is for the development of new treatments for African sleeping sickness this is not a drawback. Second, the fortuitous placement of a reactive cysteine (Cys168) near the catalytic centre offers the possibility of ‘tethering’ to assist the development of novel inhibitors (Erlanson et al., 2004). In such an approach molecular fragments that bind via a cysteine-linked intermediary are identified and subsequently embroidered in a structure-based approach to improve inhibition.

We will now seek to exploit information on known *Lm*PTR1:inhibitor complexes (Schüttelkopf *et al*., 2005) to design and test chemical entities in the search for novel lead compounds. Access to an efficient *Tb*PTR1 expression system, a reliable inhibition assay and to reproducible crystallization conditions that produce well-ordered samples will greatly assist this structure-based approach to inhibitor discovery.

## Experimental procedures

### Organisms and reagents

*Trypanosoma brucei* S427 (MITat1.4) was used as a source of genomic DNA. All routine manipulations were performed in *E. coli* strain XL-10 gold and overexpression in strain BL21(DE3) (Novagen). All chemicals were sourced from Sigma-Aldrich, BDH and CalBiochem. Restriction enzymes and DNA-modifying enzymes were from Promega or New England Biolabs.

### PCR amplification of a putative *Tb*PTR1 and cloning into pET15b

A putative sequence was identified from *T. brucei* Gene Data Bank (http://www.genedb.org) Tb927.8.2210 and an EBI mRNA sequence AF049903. Primers used to generate the full-length open reading frame by PCR were: forward (5′-CATATGATGGAAGCTCCCGCTGC-3′) containing an NdeI site and start codon, and reverse (5′-GGATCCTTAGGCATGCACAAGGCTTAAC-3′) which incorporated a BamHI site and stop codon. The resulting 0.8 kb fragment was cloned (via pCR-Blunt II-TOPO vector) into pET15b (Novagen) to generate the plasmid pET15b_*TbPTR1*. Clones were sequenced and compared with the annotated Gene Data Bank sequence and EBI mRNA sequence.

### Purification and enzyme assay

The *E. coli* strain BL21(DE3) was heat-shock transformed with pET15b-*Tb*PTR1H, which adds a histidine tag to the N-terminus of the protein product, and selected on Luria–Bertani agar plates containing ampicillin (100 mg ml^−1^). Bacteria were cultured at 37°C in SuperBroth with 100 mg ml^−1^ ampicillin to mid-log phase at which point expression of *Tb*PTR1 was induced with 1 mM isopropyl-β-d-thiogalactopyranoside and cell growth continued with vigorous aeration overnight at 25°C. Cells were harvested by centrifugation (3500 *g*, 10 min, 4°C) then resuspended in 50 mM Tris-HCl, pH 7.5, 250 mM NaCl with addition of DNase (Sigma) at 5 μg ml^−1^. The cells were lysed at a pressure of 25 Kpsi (One Shot, Constant Cell Disruptions Systems) and the extract clarified by centrifugation (30 000 *g*, 40 min, 4°C). The supernatant was filtered and applied to a 5 ml metal chelate affinity column (HiTrap; GE-Healthcare) previously charged with Ni^2+^. Unbound proteins were removed by washing with 10 column volumes of 50 mM Tris-HCl pH 7.5, containing 250 mM NaCl. The application of a 0–480 mM imidazole gradient in the same buffer, subsequently eluted His-tagged *Tb*PTR1. Fractions containing the protein were identified by SDS-PAGE, pooled and dialysed overnight against 20 mM Tris-HCl pH 7.5 and the protein concentrated to 20 mg ml^−1^. The high purity of the sample was confirmed with SDS-PAGE and matrix-assisted laser desorption/ionization time-of-flight mass spectrometry and the yield of enzyme established as approximately 25 mg l^−1^ bacterial culture. Enzyme activity was first investigated with an established spectrophotometric assay carried out at 30°C in 50 mM Na phosphate buffer, pH 6.0 (Bello *et al*., 1994).

The pH optima for substrates were determined by similar assay. Solutions containing *Tb*PTR1 (200 μg ml^−1^) and 40 μM substrate (biopterin, DHB, folate and DHF) were buffered with 20 mM sodium citrate (pH 3.0–4.5), 20 mM sodium acetate (pH 3.75–5.5) or 20 mM potassium phosphate (pH 6.0–8.0). The reaction was initiated by addition of 100 μM NADPH and the decrease in absorbance was followed at 340 nm.

*K*_m_ values for substrates were determined in a similar fashion except that biopterin and DHB concentrations varied from 1 to 80 μM, the *Tb*PTR1 concentration was 20 μg ml^−1^ and the assay was buffered with 20 mM sodium citrate, pH 3.7. Data were fitted by non-linear regression analysis using GraFit (http://www.erithacus.com/grafit/). A plot of initial velocity (*v*) versus substrate concentration of DHB (S) showed high substrate inhibition and was fitted to the following equation:

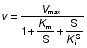

where *V*_max_, *K*_m_ and 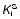 are the apparent constants for the varied substrate (S) at a fixed saturating concentration of the co-substrate NADPH (Copeland, 2000).

A dose–response curve was generated for MTX inhibition with the addition of 0.1–10 μM MTX to the assay mixture. The dose–response curve was also analysed with GraFit using Morrison's quadratic equation for tight binding inhibition (Morrison, 1969):

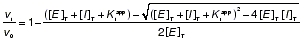

where *v*_i_ and *v*_0_ are the rates with and without inhibitor, [*E*]_T_ and [*I*]_T_ are the total concentrations of enzyme and inhibitor and 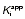 is the apparent dissociation constant for the enzyme inhibitor complex, before correction for the inhibition modality-specific influence of substrate concentration relative to *K*_m_. As MTX competes for binding with the pterin substrate, *K*_i_ can be calculated according to the equation:

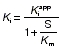

where S and *K*_m_ refer to the pterin substrate.

### Crystallographic analysis

A ternary complex of *Tb*PTR1 with cofactor and MTX was prepared by incubating PTR1 (6 mg ml^−1^), 1 mM NADP^+^, 1 mM MTX and 20 mM dithiothreitol, all in 20 mM Tris-HCl pH 7.0, on ice for 20 min and crystallization screens carried out. Hanging drops were assembled by mixing 1.5 μl of protein solution with 1.5 μl of reservoir and incubated over 100 μl of reservoir, 0.1 M sodium cacodylate pH 6.5 and 1.4 M sodium acetate. Well-ordered monoclinic blocks (0.1 mm × 0.1 mm × 0.05 mm) grew at room temperature in several days.

A crystal was briefly soaked in 30% glycerol and 70% of the reservoir solution then flashed cooled to −173°C in a stream of nitrogen gas (X-stream, Rigaku-MSC). Diffraction data were measured using a Rigaku Micromax 007 rotating anode (CuK_α_, λ = 1.5418 Å, 40 kV, 18 mA) and R-AXIS IV^++^ dual image plate detector system. Data to 2.2 Å resolution were collected using oscillations of 0.5° with an exposure time of 10 min per image, and processed using Denzo/Scalepack (Otwinowski and Minor, 1997) and CCP4 (Collaborative Computing Project Number 4, 1994) software. Five per cent of the data were flagged for the calculation of *R*_free_ to monitor refinement protocols. The crystals display space group *P*2_1_, with unit cell dimensions *a* = 74.6, *b* = 90.2, *c* = 80.8 Å, β = 115.8°. A homotetramer of total mass approximately 114 kDa constitutes the asymmetric unit.

A poly Ala model for a subunit based on *Lm*PTR1 (Gourley *et al*., 2001; Protein Data Bank code 1E92) was used in molecular replacement calculations (molrep; Vagin and Teplyakov, 2000). Four copies of this model, denoted subunits A–D, were positioned in the *Tb*PTR1 unit cell with the quaternary structure typical of many SDR family members. Following rigid body refinement (refmac5; Murshudov *et al*., 1997), the *R*_work_ was 40.1% (*R*_free_ 46.9%) and the correlation coefficient was 0.56. Rounds of restrained maximum likelihood refinement, model manipulation and graphic inspection of electron density (2*F*_*o*_*-F*_*c*_) and difference density (*F*_*o*_*-F*_*c*_) maps (*F*_*o*_ is the observed structure-factor amplitudes, *F*_*c*_ the structure-factor amplitudes calculated from the model) were carried out using refmac5 and coot (Emsley and Cowtan, 2004). The placement of ligands, water molecules and assignment of several multiple conformers completed the analysis. NCS restraints were not imposed on the model during refinement.

Several residues could not be modelled satisfactorily due to diffuse electron density. This applies to the surface loops that link β4 with α4, and α4 with β5. The residues in the first segment could not be identified in any of the four polypeptide chains of the asymmetric unit, and those from the latter segment could be modelled in subunit C only. Large positive features observed in difference density maps in the vicinity of Cys59 and Cys168, for all subunits, were compatible with covalent modification by cacodylate to form dimethylarsinoyl cysteine. Two positive difference density peaks were also observed between the His179 side-chains of subunits A and C, and chains B and D. These were modelled as Ni^2+^ and assigned occupancy of one-third. The presence of cacodylate and Ni^2+^ are artefacts of the crystallization and purification processes respectively.

## Supplementary material

The following supplementary material is available for this article online:

Fig. S1. The difference density omit map (chicken wire) for dimethylarsinoyl-modified Cys59 (top) and Cys168 (bottom) of subunit A.

This material is available as part of the online article from http://www.blackwell-synergy.com
