# Supplementary material for: Structure and reactivity of Trypanosoma brucei pteridine reductase: inhibition by the archetypal antifolate methotrexate
Source: Mol Microbiol. 2006 Aug 10;61(6):1457–68. doi: 10.1111/j.1365-2958.2006.05332.x (PMC1618733; doi:10.1111/j.1365-2958.2006.05332.x)

Supplemental Figure S1.

The difference density omit map (chicken wire) for dimethylarsinoyl-modified Cys59 (top) and Cys168 (bottom) of subunit A. The map was calculated with ($F_o$-$F_c$), $\alpha_c$ coefficients and contoured at the 3σ level; the As position gives a peak of height 16σ, a water O typically 3 or 4σ. $F_o$ represents the observed structure factors, $F_c$ the calculated structure factors and $\alpha_c$ the calculated phases. The atomic coordinates of the dimethylarsinoyl group did not contribute to $F_c$ or $\alpha_c$. Atomic positions are depicted in stick-mode colored As purple, C black, N blue, O red.

dimethylarsinoyl-Cys59

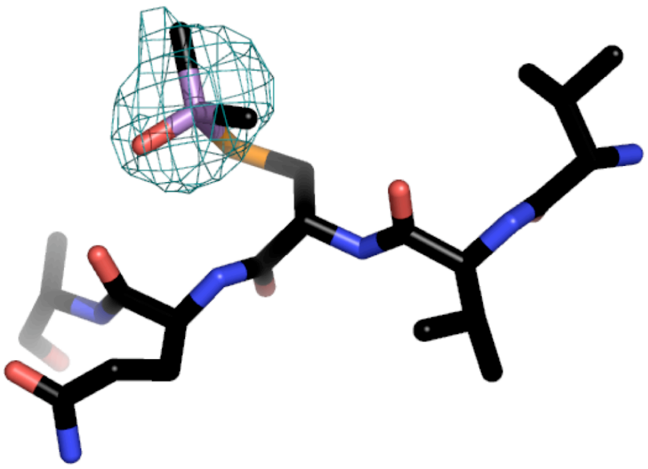

dimethylarsinoyl-Cys168

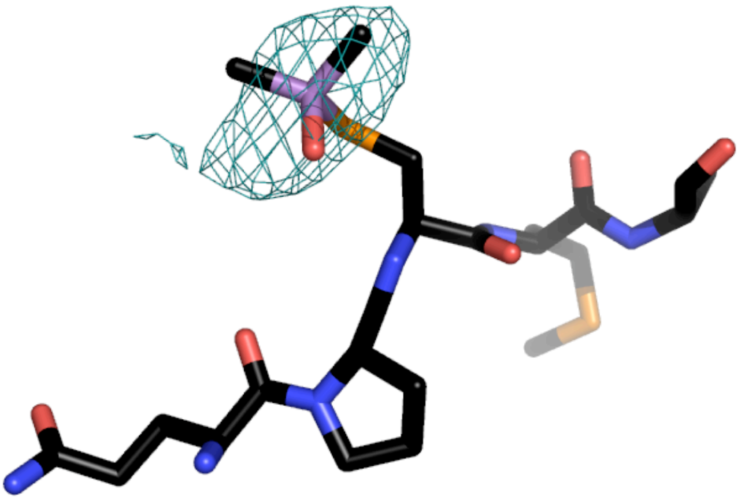

Supplement: Fig. S1. — The difference density omit map (chicken wire) for dimethylarsinoyl-modified Cys59 (top) and Cys168 (bottom) of subunit A. [file mmi0061-1457-FigS1.pdf]
